# Supplementary material for: Probing Interplays between Human XBP1u Translational Arrest Peptide and 80S Ribosome
Source: J Chem Theory Comput. 2021 Dec 9;18(3):1905–14. doi: 10.1021/acs.jctc.1c00796 (PMC8908735; doi:10.1021/acs.jctc.1c00796)
Supplement: Supplementary file 1 — ct1c00796_si_001.pdf [file ct1c00796_si_001.pdf]

SUPPORTING INFORMATION

# Probing interplays between human XBP1u translational arrest peptide and 80S ribosome

*Francesco Di Palma[1], Sergio Decherchi[1], Fátima Pardo-Avila[2], Sauro Succi[1,6,7], Michael Levitt[2], Gunnar von Heijne[*3,4], Andrea Cavalli[*1,5]*

1 Computational & Chemical Biology, Fondazione Istituto Italiano di Tecnologia, Via Morego 30, I-16163 Genova, Italy

2 Department of Structural Biology, Stanford University, Palo Alto, CA, USA, 94305

3 Department of Biochemistry and Biophysics, Stockholm University, Stockholm, Sweden

4 Science for Life Laboratories Stockholm University, Solna, Sweden

5 Department of Pharmacy and Biotechnology, University of Bologna, Via Belmeloro 6, I-40126 Bologna, Italy

6 Center for Life Nano&Neurosciences at La Sapienza, Fondazione Istituto Italiano di Tecnologia, via Regina Elena, 00161, 295, Roma, Italy

7 Physics Department, University of Harvard, Oxford Street, 29, Cambridge, USA.

KEYWORDS: Ribosome Stalling, Human XBP1u, Arrest Peptide, Unfolded Protein Response, Molecular Dynamics, Non-equilibrium Simulations.

**Supporting Information**. Detailed interaction description of the Stalled Ribosome and of the Extraction of AP variants from the exit tunnel; Energetic analysis; Force field comparison extracting AP variants from the exit tunnel: Amber vs CHARMM; Supplementary Figures including Resulting CV time-course from the 20 independent ABMD replicas for each AP variant, Trials of different force constant of the ABMD harmonic potential, Root Mean Square Deviation of the 1 μs equilibrium simulation, AP Intramolecular contacts and Intermolecular contacts between AP and ribosome over 1 μs equilibrium simulation, Comparative Q253-W256 interaction time-course from the WT and S255A variant simulations and Conserved interactions in the 100 ns ABMD simulations of the mutated residues in the WT and four AP variants; Supplementary Tables concerning AP Intramolecular and

Intermolecular AP-ribosome contacts along the 1 μs equilibrium simulation, Original inter- and intramolecular contacts average time-loss, Average detachment time-intervals $<\Delta t^i>$ along the out of equilibrium 100 ns ABMD simulations, Average Ribosome/AP non-bonded energetic contribution and Force field comparison outcomes.

**The stalled ribosome: detailed interactions**. Here we analyze the resulting interactions along the 1 μs-long (plain) molecular dynamics (MD) simulation of the stalled ribosome with the XBP1u arresting peptide (AP)[1–3] inside the exit tunnel. We start the analysis from the amino acids closest to the P-tRNA up to the terminal part of the ribosome exit tunnel. The same nomenclature of the main text (see Fig. 2) to distinguish AP residues is used: CT- for the C-terminal portion of the AP (residues 260-254); I- for the intermediate part (residues 253-247) and NT- for the N-terminal part (246-237). For the ribosome, we prepend the specific subunit name. The C-terminal CT-Met260 interacts with P-tRNA A76; this interaction was stable for the whole simulation (even without a covalent bond between them that was not included in preparation for the NC release in the Adiabatic Bias MD[4] simulation campaign). In contrast, two non-specific fluctuating contacts were observed with the backbone of 28S-C3909 and with 28S-U4531 (Supplementary Fig. 4). CT-Leu259 interacts with 28S-C4398, 28S-U4452, and 28S-U4531. The first uracil is engaged in an electrostatic interaction with the backbone nitrogen of the leucine, whereas the nucleobases of 28S-C4398 and 28S-U4531 stabilize the leucine side chain (Supplementary Fig. 4). CT-Pro258 is surrounded by 28S-U4531, 28S-G3907, and 28S-A3908 (Supplementary Fig. 4). The latter additionally stabilizes the CT-Lys257 backbone, side chain of which stacks with 28S-U4532 via a pi-cation interaction. This stacking interaction, combined with the pi-cation between the positively charged ε-amino group and the sugar of 28S-U4531, contributes to limit the movement of these PTC bases (Supplementary Fig. 4). Moreover, these C-terminal residues, in particular CT-Leu259, partially fill the space that the incoming loaded A-tRNA should occupy; indeed, the resulting complex network of interactions rigidify the structure around the PTC, preventing the ribosome cycle to move on.

CT-Ala255 interactions with 28S-U4532 and 28S-U4555 were initially absent but early established and stable, even if non-specific (Supplementary Fig. 5). CT-Pro254 was in close contact with 28S-U3644, 28S-A3908, 28S-U4552, and 28S-U4555, forming a partial stacking (Supplementary Fig. 5). 28S-U4552 is also involved in a very stable hydrogen bond with I-Gln253 reporting few fluctuations in the simulated time-scale (Supplementary Fig. 5). I-His252 showed a transient and unusual T-shaped pi-stacking interaction with uL22-His133 ribosomal protein (Supplementary Fig. 5).

The charged side-chain position of I-Arg251 was initially stabilized by a salt bridge with the phosphate of 28S-A4388; after 50 ns the salt bridge switched to the phosphate of 28S-A3908, gradually gaining a new stable configuration (Supplementary Fig. 5). This nucleobase stabilizes I-Gly250 via an H-bond that becomes transient and discontinuous in the second half of the simulation (Supplementary Fig. 5). I-Trp249 (also mentioned in the main text) side chain was persistently flanked by 28S-U4557 and at the same time obtained a transient H-bond with uL4-Arg71; this last residue lost its interaction with the backbone of NT-Pro243 (Supplementary Fig. 6).

Even if in the starting structure[3] I-Gln248, I-Cys247, and NT-Leu246 were not bearing any particular interaction (except a very unstable contact between I-Cys247 and 28S-U4555), they found pretty early some interacting partners namely uL22-Arg135, uL22-Gly134, uL22-His133, respectively (Supplementary Fig. 6). We found a very stable π-cation between NT-Phe245 and uL4-Arg71. In contrast, the initial contacts of this arginine with NT-Pro244 and NT-Pro243 (due to a rotation of 180° of the arginine $\chi_1$ dihedral angle) were almost completely lost. The π–π stacking between NT-Tyr241 and 28S-C2794 was preserved almost till the end of the simulation, becoming unstable after ~870 ns (Supplementary Fig. 6); this gave another indication about the weak nature of the contacts involving the AP amino acids that are close to the mouth of the exit channel. At variance of NT-Val239 that had no persistent contacts to mention, both NT-Pro240 and NT-Pro238 interacted with uL22-Arg128 side chain (Supplementary Fig. 6). Moreover, NT-Pro238 was also locked between the backbone of 28S-C368 and

28S-C2794 rRNA (Supplementary Fig. 6). It is finally worth mentioning that the N-terminal end of NT-Asp237 established an H-bond with the phosphate of 28S-C368 (Supplementary Fig. 6).

**The stalled ribosome: energetic analysis**. To complement the molecular interaction analysis summarized in Figure 3 we performed an energetic analysis of the 1 μs-long MD simulation. Far from being rigorous and accurate as a free-energy prediction, extracting the non-bonded and electrostatic interaction energy terms (Lennard-Jones plus Coulomb) from the plain simulation, provides a rough estimation of the per-residue contribution to the "binding" of the AP to the ribosome, as reported in Supplementary Table 5.

**Extraction of AP variants from the exit tunnel: detailed interactions.** In the C247K/S255A ABMD simulations (Supplementary Fig. 8e/f), the initial interactions lasted all the way long (Supplementary Fig. 9e). This further confirmed that C247K/S255A variant was stacked inside the channel, mainly due to the positively charged lysine that was introduced with the mutation at residue 247. This is somewhat in accordance with the conclusions in Nissley et al.,[5] in which nascent chains carrying several positive charges have been found to be ejected out of the *E. coli* ribosome exit tunnel slowly.

In the simulations with the C247S/P254C/S255A variant (Supplementary Fig. 8d), a clear pattern common to all replicas appeared: the complete solvation of the AP was prevented by prolonged interactions (more than 35 ns) that retained the C-terminal residues inside the exit channel (Supplementary Fig. 9d). These interactions were CT-Cys254 with 28S-A1600 and 28S-C2794, CT-Ala255 with uL22-Arg135, CT-Trp256 with uL4-Arg71.

Conversely, in both the S255A and the W256A variants (Supplementary Fig. 8b and c, respectively), CT-Cys254 and CT-Ala255/Ser255 could not find any stable interaction along the tunnel, thus not preventing their release into the solvent (Supplementary Fig 9b and c). Even if the transient inter-molecular interactions reported for the mutated AP residues of the variants were different, in S255A (Supplementary Fig 9b), all the key contacts with the ribosome were lost after ~30 ns of Adiabatic Bias

MD simulation.[4] In contrast, in W256A (Supplementary Fig 9c), this happened at >40 ns. Eventually, such a difference resulted in an increased total release time for the W256A variant relative to S255A (Table 1).

The behavior of the WT ABMD replicas was globally very similar to the S255A variant one. The nascent chain was released faster than any of the variants from the ribosome exit channel into the solvent (Table 1), even if its detachment was slightly slower than the W256A variant one. The pattern of interactions established by the WT residue interested by mutation in the different variants (i.e. 247, 254, 255, 256) was a kind of mix between the S255A and W256A (Supplementary Fig. 9a): NT-Cys247 and NT-W256 carried almost the same interactions as in S255A; CT-Pro254 interacted with 28S-A3908, 28S-U4555, uL22-Gly134 and uL4-His85 like in S255A, and with 28S-G4527, 28S-U4556 and 28S-C368 like in W256A; CT-Ser255 pattern of interactions was identical to the one in W256A, but the WT interactions lasted on average quite less than the ones in the W256A replicas.

We also calculated the average distance (D) covered by the WT and the different AP variants (Table 1). We obtained D = 12.5 nm ± 0.00 in both the WT and the S255A variant as they always successfully reached the end point in all the 20 replicas as defined in the ABMD protocol. For W256A, not all the replicas (15/20) reached the end point, thus $D_{W256A}$ = 11.7 nm ± 0.8, slightly below the previously mentioned distances. For the triple mutant, C247S/P254C/S255A, we calculated $D_{C247S/P254C/S255A}$ = 7.4 nm ± 2.6 with not even one replica in which the nascent chain resulted fully ejected in the solvent even if 17 of them detached from the PTC. For C247K/S255A, the distance covered was only $D_{C247K/S255A}$ = 0.47 nm ± 0.01, thus no detaching from PTC nor nascent chain ejection was possible.

**Force field comparison extracting AP variants from the exit tunnel: Amber vs CHARMM.** As reported in Supplementary Table 6, for comparative purposes, we ran 20 additional ABMD simulations on the WT and C247S/P254C/S255A variant using the CHARMM36[6,7] (July 2021 update) force field (ff), using the same ABMD protocol and parameters as in the Amber simulations (see Methods for details).

As observed with the Amber ff, the WT AP detached from the PTC and was fully extracted from the ribosomal exit tunnel in all the 20 CHARMM simulations. The average detach time and the average extraction time were respectively 1.5 and 1.3 times longer than the Amber counterparts.

The C247S/P254C/S255A variant detached in 16 over 20 CHARMM simulations (compared to 17 over 20 Amber simulations) and was not completely solvated after 100 ns in any of the CHARMM (nor Amber) simulations, and covered an average distance of 6.25 nm ± 3.25 (7.43 nm ± 2.65 for Amber). The average detach time was increased 1.9-fold with the CHARMM ff compared to the Amber ff. The relative increase in <t> for C247S/P254C/S255A compared to WT was similar for the Amber and CHARMM ff's (2.0 vs. 2.7) and the number of detached replicas was also similar (20 over 20 for WT with both ff's, 17 over 20 vs. 16/20 for C247S/P254C/S255A). Thus, the two ff's yield broadly consistent results, particularly in ranking terms.

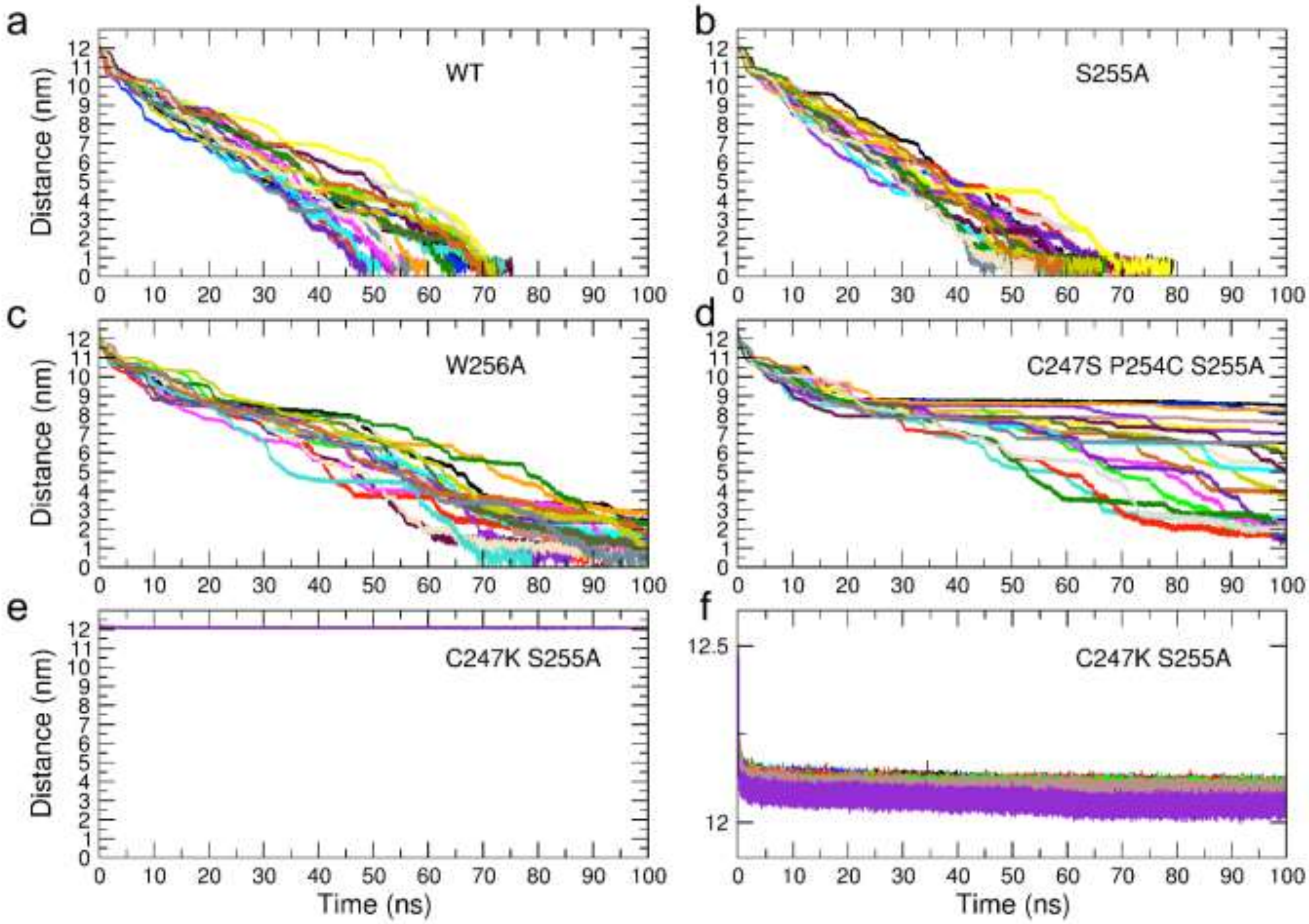


**Supplementary Figure 1.** Resulting CV time-course from the 20 independent ABMD replicas for each AP variant. In the e) panel, C247K/S255A variant, using the same y-axis interval of the other plots for comparison purpose, the 20 curves appear completely overlapped, thus, its zoom is shown in the f) panel for clarity.

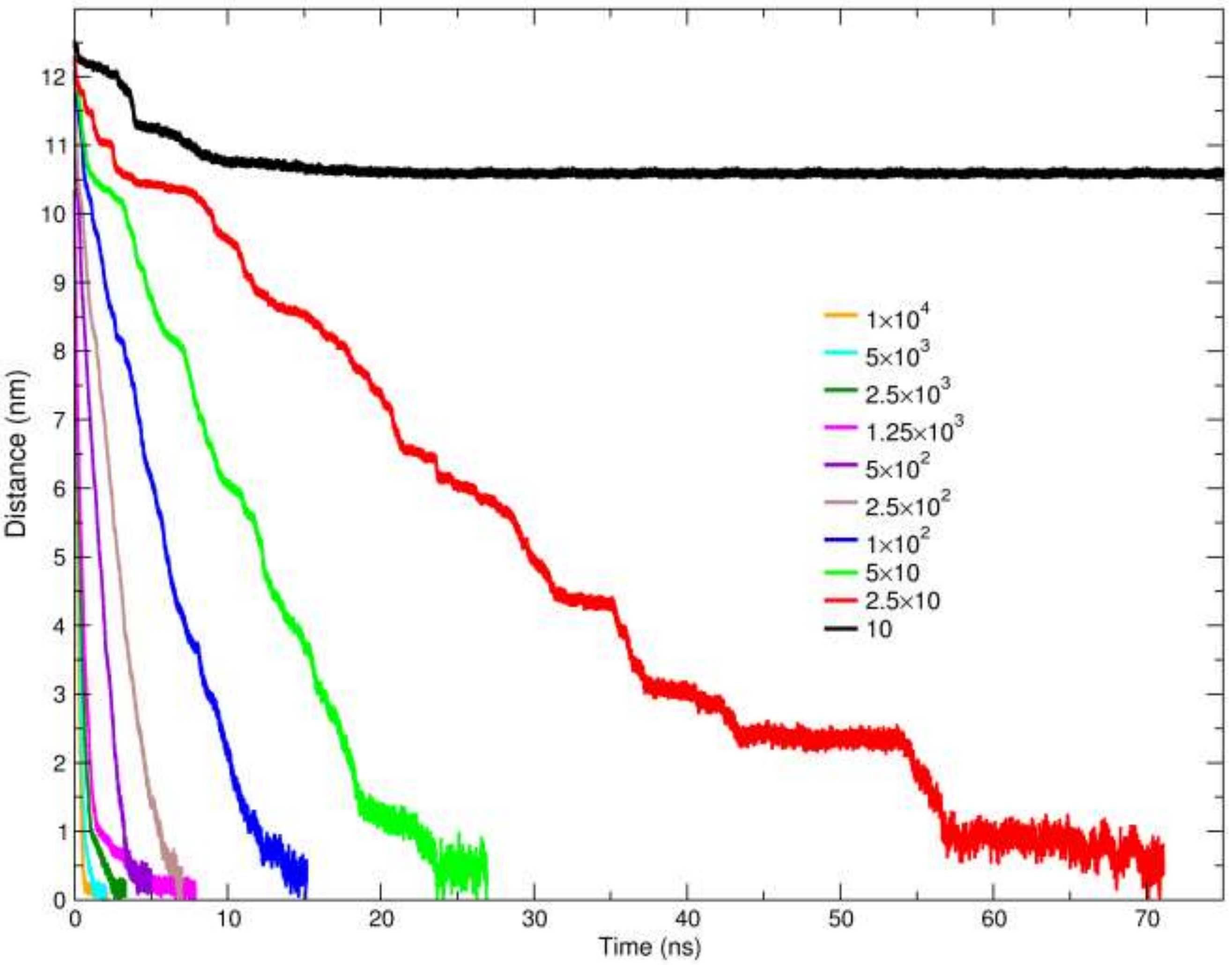


**Supplementary Figure 2.** Resulting CV time-course from nine trials of different force constant ($K$) of the ABMD harmonic potential spanning from 10 to $10^4$ $\frac{kJ/mol}{nm^4}$. In the second simulative campaign, to properly sample the intermediate states of the APs exit process, $K$ = 25 $\frac{kJ/mol}{nm^4}$ was set up.

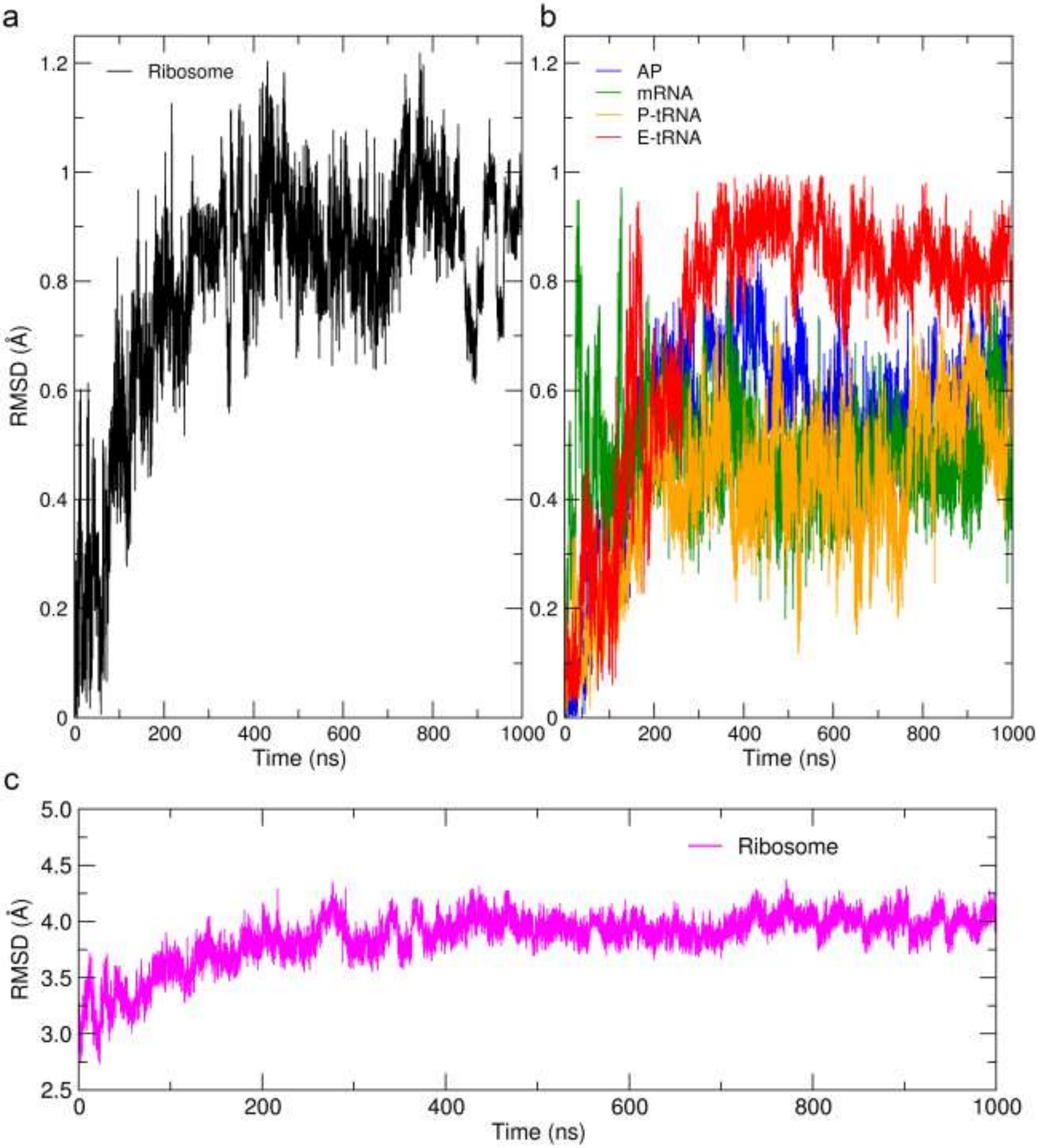


**Supplementary Figure 3.** Root Mean Square Deviation of the 1μs equilibrium simulation. a) RMSD of the sugar-phosphate and protein backbone atoms of the whole Ribosome with respect to the equilibration final conformation. b) RMSD of the backbone of AP (blue). mRNA (green). P-tRNA (Orange). E-tRNA (red) with respect to the equilibration final conformation. c) RMSD of the heavy atoms of the whole Ribosome with respect to the cryo-EM structure.

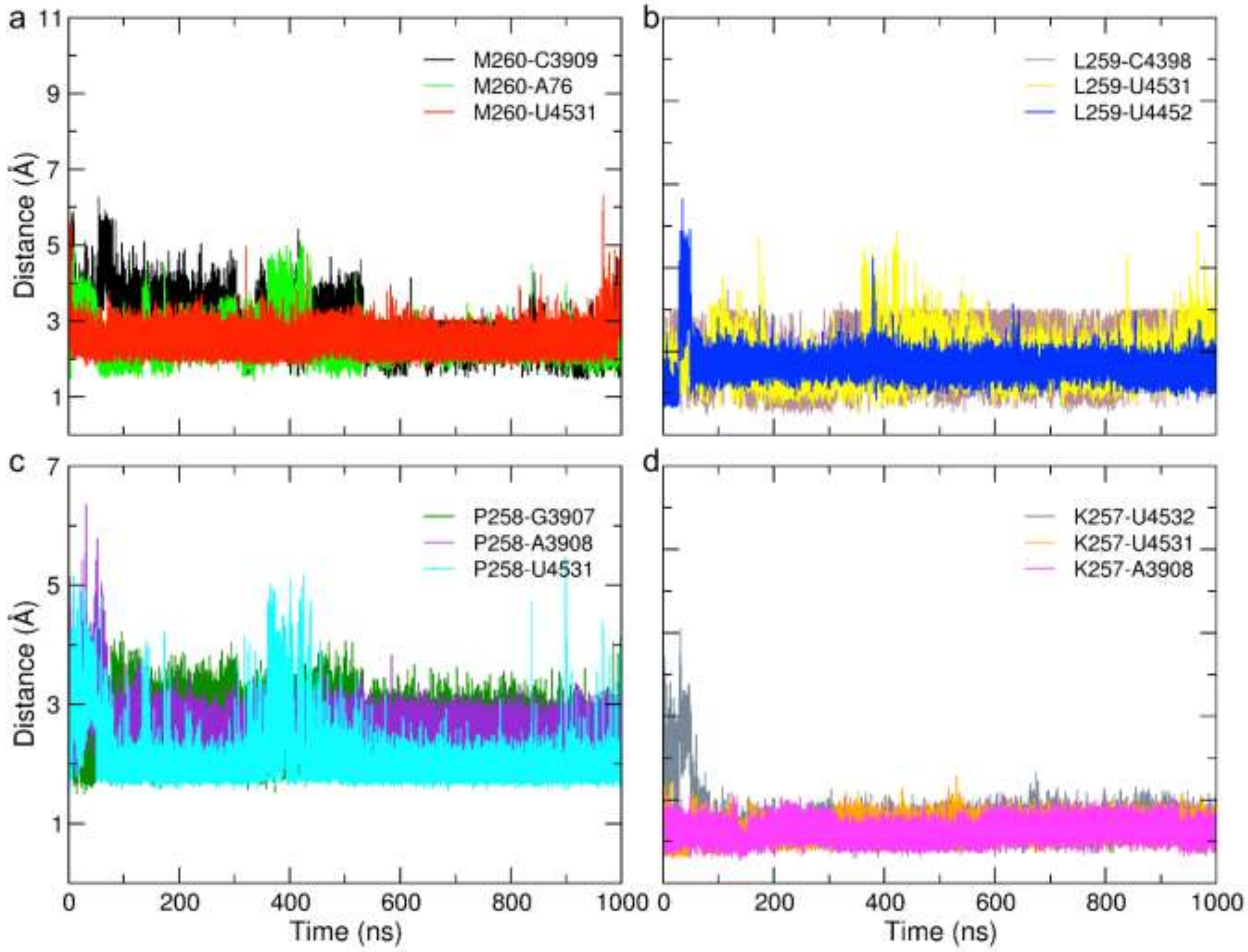


**Supplementary Figure 4.** Intermolecular contacts between AP and ribosome over 1 μs equilibrium simulation. In each graph, the following AP residues interacting with their partners can be found a) M260; b) L259; c) P258; d) K257. A76 in panel "a" is the 3' nucleobase of the P-site tRNA.

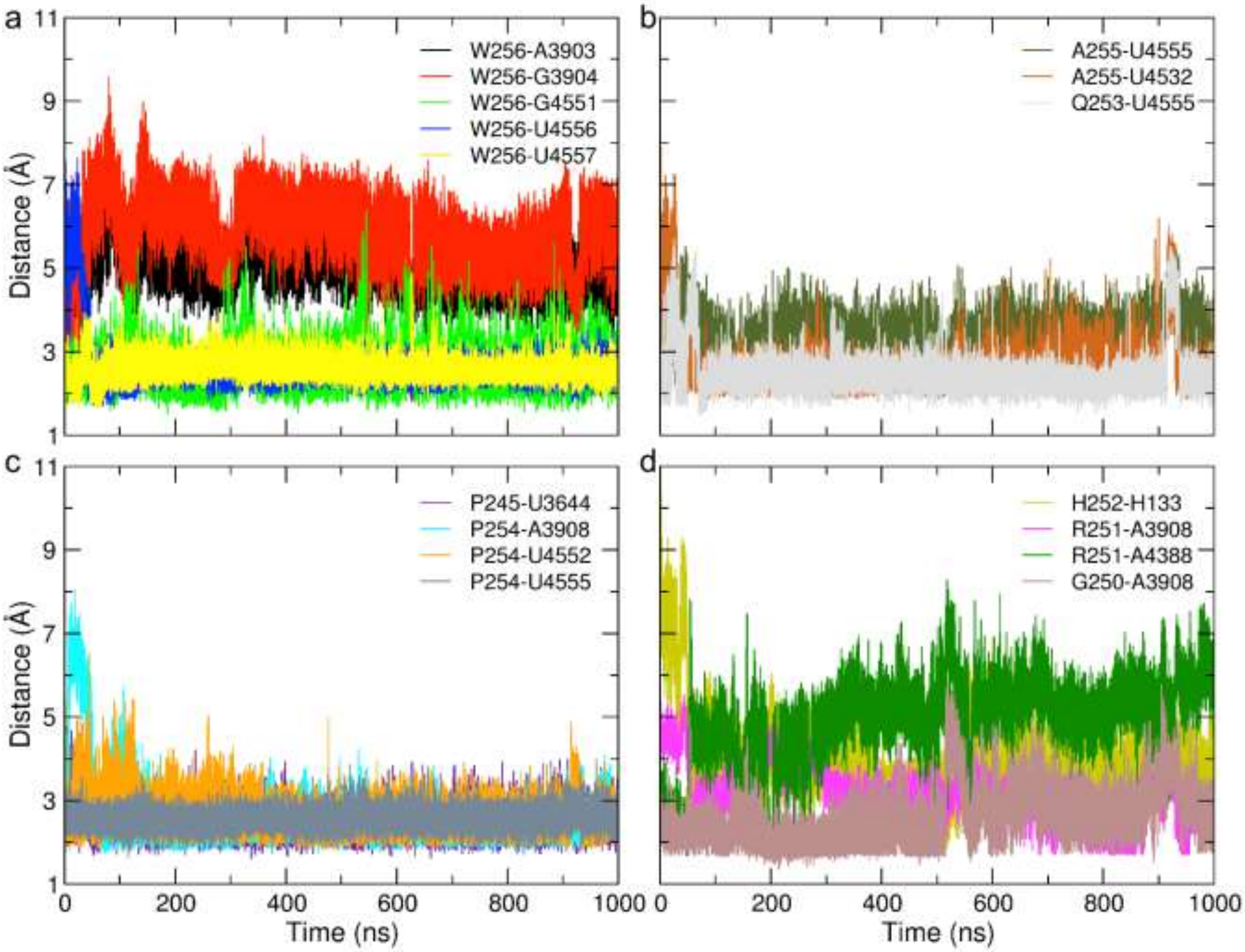


**Supplementary Figure 5.** Intermolecular contacts between AP and ribosome over 1 μs equilibrium simulation. In each graph, the following AP residues, interacting with their partners, can be found a) W256; b) A255 and Q253; c) P254; d) H252. R251 and G250.

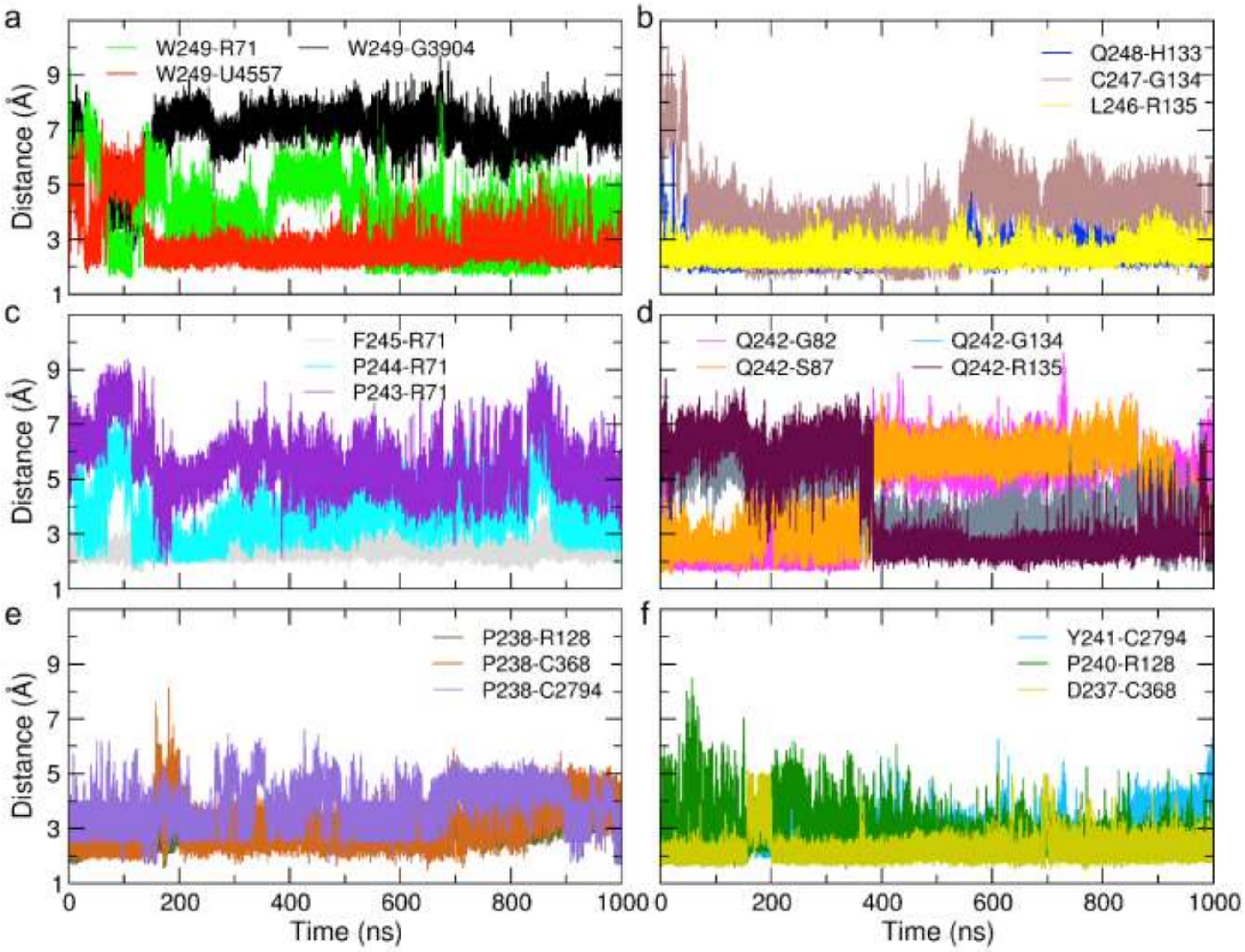


**Supplementary Figure 6.** Intermolecular contacts between AP and ribosome over 1 μs equilibrium simulation. In each graph, the following AP residues, interacting with their partners, can be found a) W249; b) Q248, C247, and L246; c) F245, P244, P243; d) Q242; e) P238; f) Y241, P240, and D237.

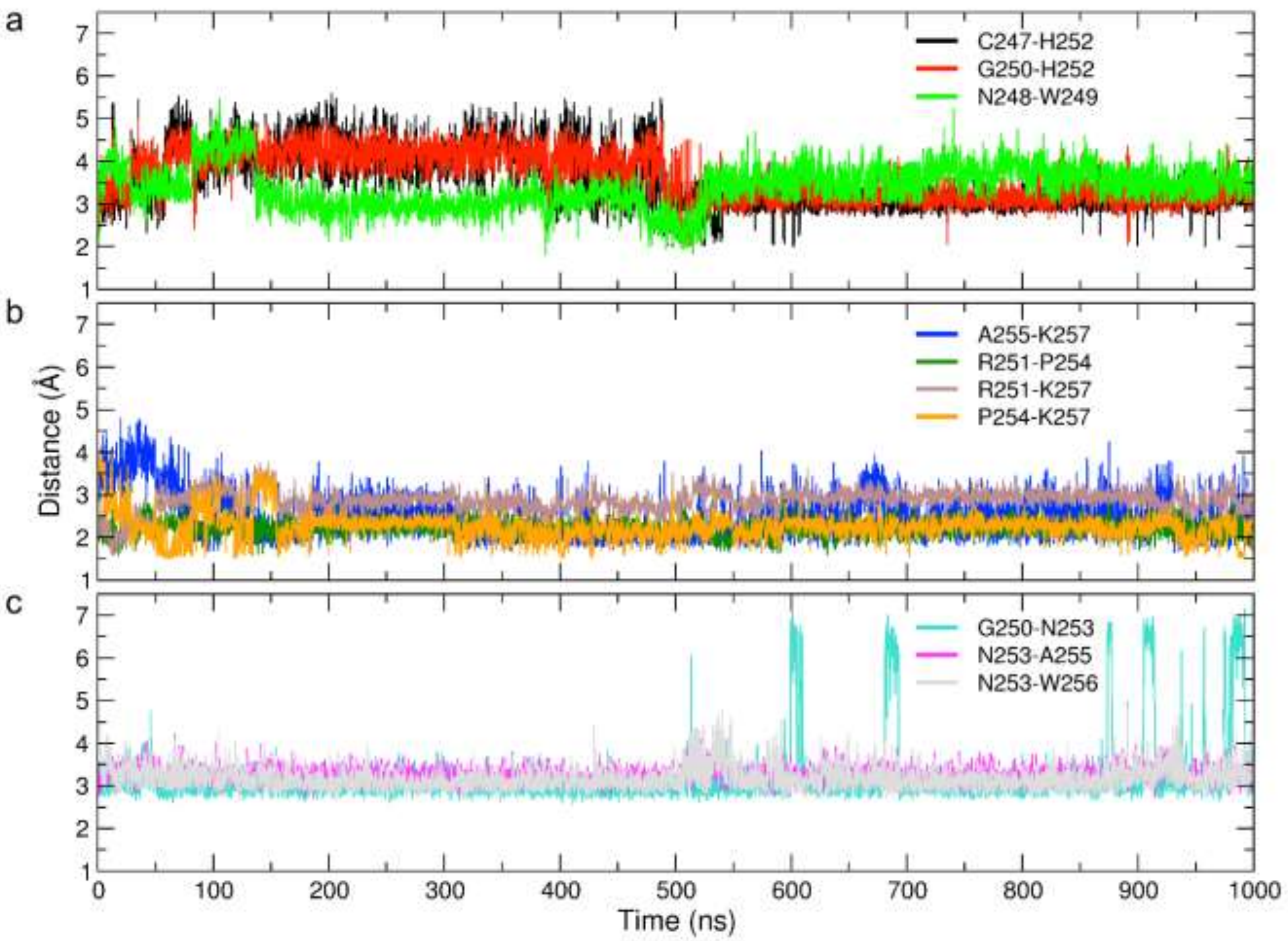


**Supplementary Figure 7.** AP intramolecular contacts over 1 μs of MD. The three networks, as described in the text are split in the 3 panels: a) C247, Q248 and W249 (CN1); b) G250, Q253, and W256 (CN2); c) R251, P254, A255, and K257 (CN3).

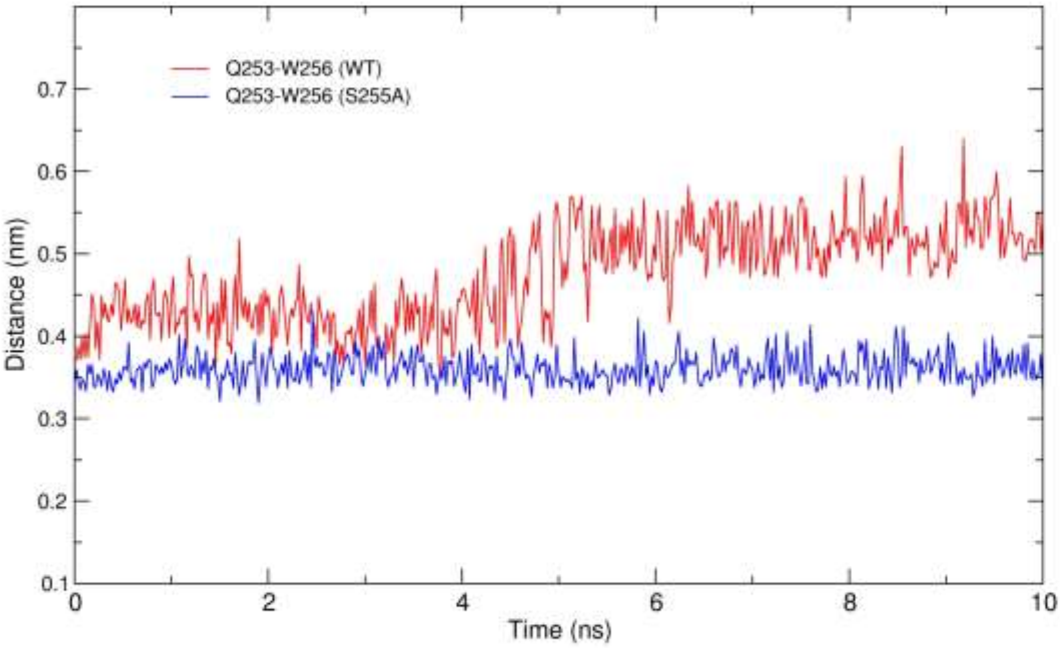


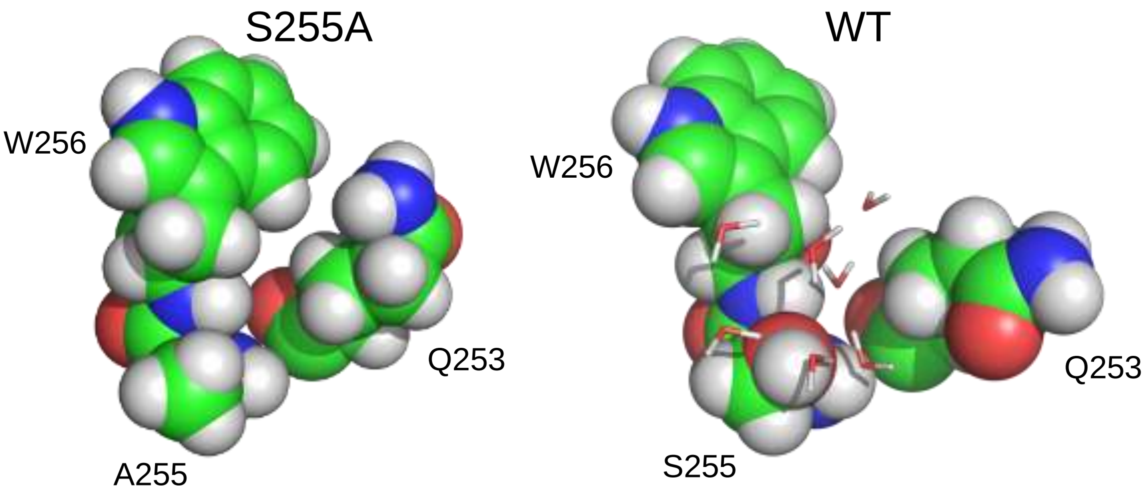


**Supplementary Figure 8.** Comparative Q253-W256 interaction time-course from the WT and S255A variant 10 ns equilibration simulations. The molecular models show the close-up on the Q253-W256 interaction in the presence of CT-A255 (left, S255A variant) and of CT-S255 (right, WT). In the latter case, the presence of several water molecules solvating serine side chain prevents an important packing interaction between Gln253 and Trp256 seen in S255A variant.

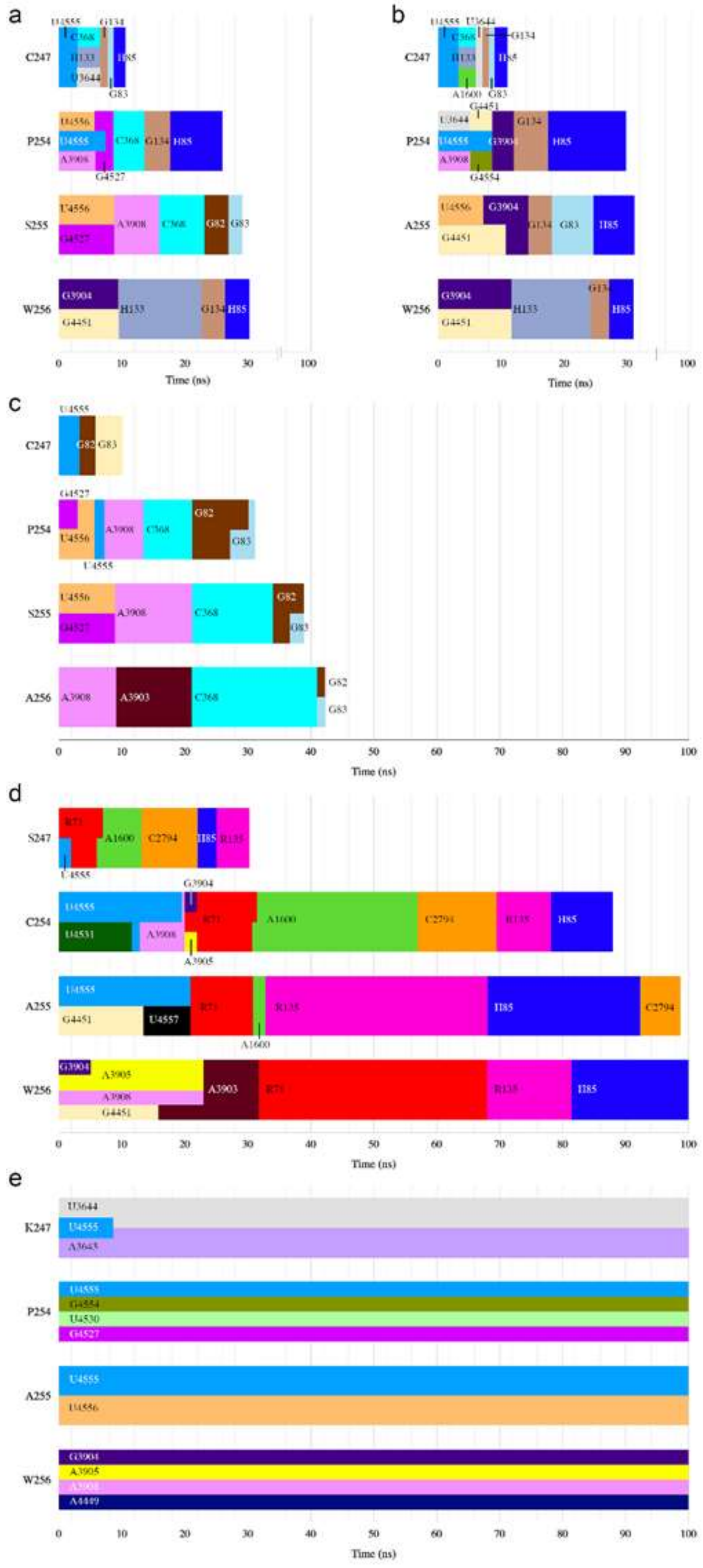

**Supplementary Figure 9.** Conserved interactions in the 100 ns ABMD simulations of the mutated residues (numbers 247, 254, 255 and 256) in the WT and four AP variants. The early part of each graph, representing the trajectories up to the point when individual residues detach from the

PTC, are the same as in Fig. 5 in the main text, while the remaining part represents the extraction phase after the detachment of Met260 from the PTC, in which the AP is pulled out of the ribosome. a) WT, b) S255A, c) W256A, d) C247S/P254C/S255A, e) C247K/S255A. For each residue the colored bar represents the range of time (in nanoseconds) in which the AP was interacting with the specific residue/nucleobase of the ribosome. The end of the bar represents the first time instant for which in at least one of the simulations the interaction was lost (the bar width does not encode any data; it is just for convenience of graphical representation).

**Supplementary Table 1**. Intermolecular AP-ribosome contacts along the equilibrium 1 μs long MD simulation.

| AP residue | Ribosomal bases/residue | Persistency | Behavior |
|---|---|---|---|
| M260 | A76 (P-tRNA) | .76 | Half fluctuating/half stable |
| " | C3909 (28S rRNA) | .67 | Bimodal |
| " | U4531 (28S rRNA) | .99 | Stable |
| L259 | U4452 (28S rRNA) | .93 | Stable |
| " | C4398 (28S rRNA) | .69 | Fluctuating |
| " | U4531 | .81 | Some fluctuations |
| P258 | G3907 (28S rRNA) | .90 | Few fluctuations |
| " | A3908 (28S rRNA) | .92 | Few initial fluctuations |
| " | U4531 | .92 | Few fluctuations |
| K257 | A3908 | 1.0 | Stable |
| " | U4531 | 1.0 | Stable |
| " | U4532 (28S rRNA) | .94 | Stable after 50 ns |
| W256 | A3903 (28S rRNA) | .03 | Lost at 35 ns |
| " | G3904 (28S rRNA) | .01 | Lost in few ns |
| " | G4551 (28S rRNA) | .96 | Stable |
| " | U4556 (28S rRNA) | .96 | Gained in few ns |
| " | U4557 (28S rRNA) | .99 | Stable |

| | | | |
|---|---|---|---|
| A255 | U4532 | .90 | Gained at 35 ns |
| " | U4555 (28S rRNA) | .30 | High Fluctuations |
| " | U4552 (28S rRNA) | .97 | Stable |
| P254 | U3644 (28S rRNA) | .93 | Stable |
| " | A3908 | .90 | Stable |
| " | U4555 | .85 | Initial fluctuation |
| Q253 | U4555 | .93 | Few fluctuations |
| H252 | H133 (uL22) | .32 | Gained at 50ns/transient |
| " | A4388 (28S rRNA) | .08 | Lost after 50 ns |
| R251 | A3908 | .71 | Gained at 60ns/fluctuating |
| G250 | A3908 | .76 | Transient after 500ns |
| W249 | G3904 | .01 | Lost in few ns |
| " | U4557 | .87 | Quite Stable |
| " | R71 (uL4) | .42 | Gained at 70 ns/transient |
| Q248 | H133 (uL22) | .94 | Stably gained at 25ns |
| C247 | U4555 | .08 | Definitely lost after 100ns |
| C247 | G134 (uL22) | .48 | Gained at 50ns/transient |
| L246 | R135 (uL22) | .94 | Stably Gained in few ns |
| F245 | R71 | .97 | Stable |
| P244 | R71 | .32 | Highly Fluctuating |
| P243 | R71 | .02 | Lost in few ns |

| | | | |
|---|---|---|---|
| Q242 | G82 (uL4) | .34 | Lost at 400ns |
| “ | S87 (uL4) | .31 | Lost at 400ns |
| “ | G134 | .40 | Gained at 400ns |
| “ | R135 | .59 | Gained at 400ns |
| Y241 | C2794 (28S rRNA) | .88 | Stable till 870 ns |
| P240 | R128 (uL22) | .87 | Almost stable |
| P238 | R128 | .59 | Bimodal |
| “ | C368 (28S rRNA) | .85 | Stable till 800ns |
| “ | C2794 | .56 | Bimodal |
| D237 | C368 | .94 | Barely Fluctuating |

**Supplementary Table 2.** Intramolecular AP-AP contacts along the equilibrium 1 μs long all-atom MD simulation.

| AP residue 1 | AP residue 2 | Persistency | Behavior |
|---|---|---|---|
| C247 | H252 | .56 | First half fluctuating/ second half very stable |
| Q248 | W249 | .89 | Short fluctuations after 100 ns |
| G250 | H252 | .55 | First half fluctuating/ second half very stable |
| R251 | P254 | 1.0 | Stable |
| R251 | K257 | .99 | Stable |
| P254 | K257 | .88 | Few initial fluctuations |
| A255 | K257 | .80 | Initially fluctuating, quite stable after 150 ns |
| Q253 | G250 | .92 | Short fluctuations after 500 ns |
| Q253 | A255 | .98 | Stable |
| Q253 | W256 | .97 | Stable |

**Supplementary Table 3.** Original inter- and intramolecular contacts average time-loss (in ns), and associated standard error, along the out of equilibrium 100 ns ABMD[4] simulations (as plotted in Fig. 4a). An asterisk means that the corresponding residue never lost its contacts.

| AP residue | WT | S255A | W256A | C247S/P254C/S255A | C247K/S255A |
|---|---|---|---|---|---|
| 237 | 0.20 ± 0.01 | 0.22 ± 0.01 | 0.23 ± 0.01 | 0.28 ± 0.01 | 0.26 ± 0.01 |
| 238 | 0.21 ± 0.01 | 0.25 ± 0.01 | 0.25 ± 0.01 | 0.30 ± 0.01 | 0.30 ± 0.01 |
| 239 | 0.22 ± 0.02 | 0.25 ± 0.01 | 0.26 ± 0.01 | 0.31 ± 0.01 | 0.35 ± 0.01 |
| 240 | 0.95 ± 0.04 | 1.03 ± 0.03 | 1.11 ± 0.04 | 1.25 ± 0.05 | 6.12 ± 4.72 |
| 241 | 1.33 ± 0.02 | 1.59 ± 0.04 | 1.61 ± 0.05 | 1.72 ± 0.07 | 25.18 ± 10.07 |
| 242 | 1.38 ± 0.04 | 1.60 ± 0.05 | 1.64 ± 0.05 | 1.75 ± 0.10 | * |
| 243 | 1.53 ± 0.02 | 1.78 ± 0.04 | 1.77 ± 0.05 | 1.83 ± 0.11 | * |
| 244 | 1.55 ± 0.05 | 1.80 ± 0.06 | 1.79 ± 0.06 | 1.99 ± 0.19 | * |
| 245 | 1.60 ± 0.06 | 1.85 ± 0.05 | 1.83 ± 0.10 | 2.02 ± 0.22 | * |
| 246 | 1.79 ± 0.11 | 2.05 ± 0.13 | 2.01 ± 0.21 | 3.46 ± 0.42 | * |
| 247 | 2.95 ± 0.25 | 3.22 ± 0.25 | 3.19 ± 0.30 | 7.13 ± 0.38 | * |
| 248 | 2.99 ± 0.36 | 3.25 ± 0.37 | 3.25 ± 0.34 | 8.11 ± 0.43 | * |
| 249 | 4.30 ± 0.42 | 4.57 ± 0.40 | 4.55 ± 0.51 | 9.38 ± 0.59 | * |
| 250 | 4.66 ± 0.39 | 4.96 ± 0.36 | 4.90 ± 0.47 | 11.05 ± 1.23 | * |
| 251 | 5.29 ± 0.60 | 5.67 ± 0.59 | 5.58 ± 0.70 | 12.29 ± 1.18 | * |
| 252 | 5.33 ± 0.65 | 5.67 ± 0.61 | 5.60 ± 0.66 | 13.97 ± 1.46 | * |
| 253 | 5.87 ± 0.69 | 6.45 ± 0.69 | 6.17 ± 0.72 | 15.06 ± 2.09 | * |
| 254 | 7.72 ± 0.80 | 8.66 ± 0.85 | 8.10 ± 0.81 | 19.59 ± 3.13 | * |

| | | | | | |
|---|---|---|---|---|---|
| 255 | 8.81 ± 0.88 | 10.74 ± 0.92 | 8.89 ± 0.98 | 20.84 ± 2.97 | * |
| 256 | 9.55 ± 0.91 | 11.59 ± 0.88 | 9.15 ± 0.92 | 23.03 ± 3.56 | * |
| 257 | 10.53 ± 0.78 | 12.48 ± 0.81 | 10.47 ± 1.03 | 24.15 ± 5.02 | * |
| 258 | 11.49 ± 0.90 | 13.31 ± 0.97 | 11.34 ± 1.10 | 25.31 ± 4.86 | * |
| 259 | 12.55 ± 0.92 | 13.67 ± 1.01 | 12.45 ± 1.14 | 26.14 ± 5.23 | * |
| 260 | 13.16 ± 0.89 | 13.99 ± 0.94 | 13.02 ± 1.09 | 27.12 ± 5.61 | * |

**Supplementary Table 4.** Average time-intervals <$\Delta t^i$> (in ns), and associated standard error, required to detach each AP residue after the previous one has detached during the 100 ns ABMD[4] simulations (as plotted in Fig. 4b). A star symbol means that the corresponding residue never detached.

| AP residue | WT | S255A | W256A | C247S/P254C/S255A | C247K/S255A |
|---|---|---|---|---|---|
| 237 | 0.20 ± 0.01 | 0.22 ± 0.01 | 0.23 ± 0.01 | 0.28 ± 0.01 | 0.26 ± 0.01 |
| 238 | 0.01 ± 0.01 | 0.03 ± 0.01 | 0.02 ± 0.01 | 0.02 ± 0.01 | 0.30 ± 0.01 |
| 239 | 0.01 ± 0.01 | 0.01 ± 0.01 | 0.01 ± 0.01 | 0.01 ± 0.01 | 0.35 ± 0.01 |
| 240 | 0.73 ± 0.04 | 0.77 ± 0.02 | 0.85 ± 0.03 | 0.94 ± 0.05 | 5.77 ± 3.14 |
| 241 | 0.58 ± 0.02 | 0.56 ± 0.05 | 0.51 ± 0.03 | 0.47 ± 0.07 | 19.06 ± 7.12 |
| 242 | 0.05 ± 0.01 | 0.01 ± 0.01 | 0.03 ± 0.03 | 0.03 ± 0.02 | * |
| 243 | 0.16 ± 0.02 | 0.18 ± 0.03 | 0.13 ± 0.03 | 0.08 ± 0.03 | * |
| 244 | 0.02 ± 0.01 | 0.02 ± 0.01 | 0.02 ± 0.01 | 0.16 ± 0.04 | * |
| 245 | 0.05 ± 0.02 | 0.05 ± 0.04 | 0.04 ± 0.01 | 0.12 ± 0.02 | * |
| 246 | 0.20 ± 0.05 | 0.20 ± 0.09 | 0.18 ± 0.04 | 1.44 ± 0.12 | * |
| 247 | 1.17 ± 0.08 | 1.17 ± 0.07 | 1.18 ± 0.11 | 3.67 ± 0.27 | * |
| 248 | 0.04 ± 0.01 | 0.03 ± 0.02 | 0.06 ± 0.04 | 0.98 ± 0.30 | * |
| 249 | 1.31 ± 0.22 | 1.32 ± 0.18 | 1.30 ± 0.36 | 1.27 ± 0.42 | * |
| 250 | 0.37 ± 0.04 | 0.39 ± 0.05 | 0.35 ± 0.03 | 1.67 ± 0.46 | * |
| 251 | 0.64 ± 0.12 | 0.71 ± 0.11 | 0.68 ± 0.15 | 1.24 ± 0.28 | * |
| 252 | 0.03 ± 0.01 | 0.01 ± 0.01 | 0.02 ± 0.01 | 1.68 ± 0.36 | * |
| 253 | 0.54 ± 0.13 | 0.77 ± 0.08 | 0.57 ± 0.10 | 1.09 ± 0.27 | * |
| 254 | 1.86 ± 0.16 | 2.21 ± 0.20 | 1.93 ± 0.17 | 4.53 ± 0.81 | * |

| | | | | | |
|---|---|---|---|---|---|
| 255 | 1.11 ± 0.16 | 2.08 ± 0.18 | 0.79 ± 0.19 | 1.25 ± 0.19 | * |
| 256 | 0.84 ± 0.12 | 0.85 ± 0.12 | 0.26 ± 0.10 | 2.19 ± 0.52 | * |
| 257 | 0.93 ± 0.18 | 0.89 ± 0.17 | 1.30 ± 0.22 | 1.12 ± 0.22 | * |
| 258 | 0.92 ± 0.22 | 0.83 ± 0.18 | 0.85 ± 0.20 | 1.16 ± 0.26 | * |
| 259 | 0.86 ± 0.12 | 0.36 ± 0.06 | 1.11 ± 0.14 | 0.83 ± 0.13 | * |
| 260 | 0.59 ± 0.10 | 0.32 ± 0.05 | 0.58 ± 0.09 | 0.98 ± 0.11 | * |

**Supplementary Table 5**. AP per-residue time averaged non-bonded energetic contributions during the equilibrium 1 μs long all-atom MD simulation. A star symbol means that the corresponding residue did not contribute to any intramolecular interaction.

| AP residue | Intermolecular contribution (kJ/mol) | Intramolecular contribution (kJ/mol) |
|---|---|---|
| 237 | -6.35 ± 0.32 | * |
| 238 | -6.09 ± 0.70 | * |
| 239 | -0.72 ± 0.54 | * |
| 240 | -9.02 ± 0.30 | * |
| 241 | -6.85 ± 0.52 | * |
| 242 | -19.33 ± 0.88 | * |
| 243 | -1.21 ± 0.22 | * |
| 244 | -1.17 ± 0.25 | * |
| 245 | -5.57 ± 0.71 | * |
| 246 | -9.00 ± 0.68 | * |
| 247 | -1.28 ± 0.37 | -4.05 ± 0.45 |
| 248 | -7.10 ± 0.42 | -12.52 ± 2.15 |
| 249 | -7.83 ± 0.57 | -12.52 ± 2.15 |
| 250 | -3.97 ± 0.34 | -2.15 ± 0.12 |
| 251 | -2.52 ± 0.18 | -5.36 ± 0.65 |
| 252 | -2.97 ± 1.60 | -2.7 ± 0.16 |
| 253 | -10.58 ± 0.08 | -8.79 ± 0.91 |
| 254 | -53.71 ± 3.01 | -6.72 ± 0.76 |

| | | |
|---|---|---|
| 255 | -8.57 ± 0.59 | -5.23 ± 0.53 |
| 256 | -27.69 ± 1.54 | -5.66 ± 0.54 |
| 257 | -36.98 ± 1.57 | -8.76 ± 1.64 |
| 258 | -34.98 ± 1.62 | * |
| 259 | -23.11 ± 0.75 | * |
| 260 | -30.72 ± 0.91 | * |

**Supplementary Table 6**. Force field comparison extracting WT and C247S/P254C/S255A APs from the exit tunnel via ABMD: Amber vs CHARMM.

| | AP variant | Met260 detach time from PTC $(\langle t\rangle_{M260})$ | AP extraction Time (<T>) | #Detached replicas | #Extracted replicas | Covered distance (D) in CV space |
|---|---|---|---|---|---|---|
| Amber14sb[8] + bsc0[9] + $\chi_{OL3}$[10] | WT | 13.2 ns ± 0.4 | 47.2 ns ± 1.3 | 20/20 | 20/20 | 12.5 nm ± 0.00 |
| | C247S/P254C/S255A | 27.1 ns ± 5.6 | No extraction | 17/20 | 0/20 | 7.43 nm ± 2.65 |
| Charmm36[6,7] (Jul21) | WT | 20.0 ns ± 4.3 | 60.3 ns ± 7.2 | 20/20 | 20/20 | 12.5 nm ± 0.00 |
| | C247S/P254C/S255A | 53.0 ns ± 10.2 | No extraction | 16/20 | 0/20 | 6.25 nm ± 3.25 |

**Corresponding Authors**

* **Andrea Cavalli** - Computational & Chemical Biology, Fondazione Istituto Italiano di Tecnologia, Genova I-16163, Italy; Email: andrea.cavalli@iit.it

* **Gunnar von Heijne** - Department of Biochemistry and Biophysics, Stockholm University, Stockholm SE-106 91, Sweden; Email: gunnar.von.heijne@dbb.su.se

ABBREVIATIONS

AP, arrest peptide; NC, nascent chain; PTC, polypeptide transferase center; MD, molecular dynamics; ABMD, adiabatic bias molecular dynamics; CV, collective variable.

REFERENCES

(1) Yoshida, H.; Matsui, T.; Yamamoto, A.; Okada, T.; Mori, K. XBP1 MRNA Is Induced by ATF6 and Spliced by IRE1 in Response to ER Stress to Produce a Highly Active Transcription Factor. *Cell* **2001**, *107* (7), 881–891. https://doi.org/10.1016/S0092-8674(01)00611-0.

(2) Yanagitani, K.; Imagawa, Y.; Iwawaki, T.; Hosoda, A.; Saito, M.; Kimata, Y.; Kohno, K. Cotranslational Targeting of XBP1 Protein to the Membrane Promotes Cytoplasmic Splicing of Its Own MRNA. *Mol. Cell* **2009**, *34* (2), 191–200. https://doi.org/10.1016/j.molcel.2009.02.033.

(3) Shanmuganathan, V.; Schiller, N.; Magoulopoulou, A.; Cheng, J.; Braunger, K.; Cymer, F.; Berninghausen, O.; Beatrix, B.; Kohno, K.; von Heijne, G.; Beckmann, R. Structural and Mutational Analysis of the Ribosome-Arresting Human XBP1u. *eLife* **2019**, *8*, e46267. https://doi.org/10.7554/eLife.46267.

(4) Marchi, M.; Ballone, P. Adiabatic Bias Molecular Dynamics: A Method to Navigate the Conformational Space of Complex Molecular Systems. *J. Chem. Phys.* **1999**, *110* (8), 3697–3702. https://doi.org/10.1063/1.478259.

(5) Nissley, D. A.; Vu, Q. V.; Trovato, F.; Ahmed, N.; Jiang, Y.; Li, M. S.; O'Brien, E. P. Electrostatic Interactions Govern Extreme Nascent Protein Ejection Times from Ribosomes and Can Delay Ribosome Recycling. *J. Am. Chem. Soc.* **2020**, *142* (13), 6103–6110. https://doi.org/10.1021/jacs.9b12264.

(6) Huang, J.; Rauscher, S.; Nawrocki, G.; Ran, T.; Feig, M.; de Groot, B. L.; Grubmüller, H.; MacKerell, A. D. CHARMM36m: An Improved Force Field for Folded and Intrinsically Disordered Proteins. *Nat. Methods* **2017**, *14* (1), 71–73. https://doi.org/10.1038/nmeth.4067.

(7) Denning, E. J.; Priyakumar, U. D.; Nilsson, L.; Mackerell Jr., A. D. Impact of 2′-Hydroxyl Sampling on the Conformational Properties of RNA: Update of the CHARMM All-Atom Additive Force Field for RNA. *J. Comput. Chem.* **2011**, *32* (9), 1929–1943. https://doi.org/10.1002/jcc.21777.

(8) Maier, J. A.; Martinez, C.; Kasavajhala, K.; Wickstrom, L.; Hauser, K. E.; Simmerling, C. Ff14SB: Improving the Accuracy of Protein Side Chain and Backbone Parameters from

Ff99SB. *J. Chem. Theory Comput.* **2015**, *11* (8), 3696–3713. https://doi.org/10.1021/acs.jctc.5b00255.

(9) Pérez, A.; Marchán, I.; Svozil, D.; Sponer, J.; Cheatham, T. E.; Laughton, C. A.; Orozco, M. Refinement of the AMBER Force Field for Nucleic Acids: Improving the Description of α/γ Conformers. *Biophys. J.* **2007**, *92* (11), 3817–3829. https://doi.org/10.1529/biophysj.106.097782.

(10) Zgarbová, M.; Otyepka, M.; Šponer, J.; Mládek, A.; Banáš, P.; Cheatham, T. E.; Jurečka, P. Refinement of the Cornell et al. Nucleic Acids Force Field Based on Reference Quantum Chemical Calculations of Glycosidic Torsion Profiles. *J. Chem. Theory Comput.* **2011**, *7* (9), 2886–2902. https://doi.org/10.1021/ct200162x.
